# Effectiveness of service screening: a case–control study to assess breast cancer mortality reduction

**DOI:** 10.1038/sj.bjc.6604532

**Published:** 2008-07-29

**Authors:** D Puliti, G Miccinesi, N Collina, V De Lisi, M Federico, S Ferretti, A C Finarelli, F Foca, L Mangone, C Naldoni, M Petrella, A Ponti, N Segnan, A Sigona, M Zarcone, M Zorzi, M Zappa, E Paci, the IMPACT Working Group

**Affiliations:** 1 Clinical and Descriptive Epidemiology Unit, CSPO, Research Institute of the Tuscany Region, via San Salvi 12, Florence 50135, Italy; 2 AUSL Bologna, Via del Seminario 1, S.Lazzaro di Savena, Bologna 40068, Italy; 3 Parma Cancer Registry, via Abbeveratoia 4, Parma 43100, Italy; 4 Modena Cancer Registry, via del Pozzo 71, Modena 41100, Italy; 5 Ferrara Cancer Registry, via Fossato di Mortara 64b, Ferrara 44100, Italy; 6 Emilia-Romagna Region Health Department, viale Aldo Moro 21, Bologna 40127, Italy; 7 Romagna Cancer Registry, via Carlo Forlanini 34, Forlì 47100, Italy; 8 Reggio Emilia Cancer Registry, via Amendola 2, Reggio Emilia 42100, Italy; 9 Epidemiology Unit ASL2, via XIV Settembre 79, Perugia 06100, Italy; 10 Epidemiology Unit, CPO Piemonte, via S. Francesco da Paola 31, Torino 10123, Italy; 11 Cancer Registry, A.O. ‘Civile M.P. Arezzo’, via Dante 109, Ragusa 97100, Italy; 12 Palermo Breast Cancer Registry, Piazzale N. Leotta 2, Palermo 90127, Italy; 13 Venetian Tumour Registry, Istituto Oncologico Veneto, via Gattamelata 64, Padua 35128, Italy

**Keywords:** breast cancer, case–control study, service screening

## Abstract

The aim of this study was the evaluation of the impact of service screening programmes on breast cancer mortality in five regions of Italy. We conducted a matched case–control study with four controls for each case. Cases were defined as breast cancer deaths occurred not later than 31 December 2002. Controls were sampled from the local municipality list and matched by date of birth. Screening histories were assessed by the local, computerised, screening database and subjects were classified as either invited or not-yet-invited and as either screened or unscreened. There were a total of 1750 breast cancer deaths within the 50 to 74-year-old breast cancer cases and a total of 7000 controls. The logistic conditional estimate of the cumulative odds ratios comparing invited with not-yet-invited women was 0.75 (95% CI: 0.62–0.92). Restricting the analyses to invited women, the odds ratio of screened to never-respondent women corrected for self-selection bias was 0.55 (95% CI: 0.36–0.85). The introduction of breast cancer screening programmes in Italy is associated with a reduction in breast cancer mortality attributable to the additional impact of service screening over and above the background access to mammography.

Breast cancer screening has been shown in several randomised clinical trials (RCTs) conducted between the mid-1960s and mid-1980s to be effective in reducing breast cancer mortality (IARC, 2002).

Mammographic screening is now widespread in western countries but access modalities differ. In the United States, the use of screening is based on spontaneous access to mammographic facilities, usually associated with a clinical breast examination and with different guidelines on age range and screening interval (Smith-Bindman *et al*, 2003; Smith *et al*, 2007). In Europe service screening is a public health initiative, offering two-views mammography every 2 years, in which all women of the target population (in Italy, 50–69 years of age) are invited to participate (Perry *et al*, 2006). Programme performance indicators have been developed in accordance with European guidelines and assessments have been reported (Lynge *et al*, 2003; Giordano *et al*, 2006). The first screening programme in Italy was started at the beginning of the 1990s in the cities of Florence and Turin, and since then other programmes have been started in other regions. In 2006 the National Centre for Screening Monitoring reported a coverage of more than 90% of the target population in northern and central Italy (Zappa and Rosselli Del Turco, 2007).

Now that screening is widespread, two questions become relevant: (a) are the effects of breast cancer screening under usual conditions within the community comparable with those detected by the RCTs (Harris, 2005), and (b) is service screening able to have an additional impact over and above the background, spontaneous use of mammography in the population?

There are several methods to assess the effectiveness of screening for reducing breast cancer mortality. In particular, incidence-based mortality and geographical comparisons have been used (Paci *et al*, 2002; Duffy *et al*, 2002a; Olsen *et al*, 2007). However, the case–control study is a traditional tool for the evaluation of screening outcome (Collette *et al*, 1984; Verbeek *et al*, 1984; Palli *et al*, 1986), although with high methodological complexity (Walter, 2003; Fielder *et al*, 2004; Elmore *et al*, 2005), and was used in several studies at the end of the eighties. The case–control study design has been used because of its efficiency. The collection of screening histories of a limited number of controls allows a more accurate and valid evaluation than it is possible for the whole invited or screened population. Several statistical simulations, like the one by Connor *et al*, (2000), have shown that, with adequate study design, the case–control study results are comparable to the observed in prospective cohort studies. Estimates of mortality reduction from randomised trials and case–control studies have been compared in review (Demissie *et al*, 1998).

The aim of this case–control study has been to evaluate the effectiveness of service screening programmes in reducing breast cancer mortality in the Italian areas participating in the IMPACT study. The outcome is interpreted as the additional impact of the organised programme offered to the community over and above the usual care in that population, that is, the existing, background access of target women to mammography.

## Materials and methods

### The IMPACT study

The IMPACT database (Zorzi *et al*, 2006) was collected from cancer registries data of *in situ* and invasive breast cancer cases diagnosed between 1988 and 2001 in women aged 40–79, resident in 17 areas mainly located in central and northern Italy. All cases were followed up for living status and the specific cause of death was obtained from the regional mortality registers. All cases were linked to the screening files and classified by detection method as either screen-detected, not screen-detected but with at least one screening test, never-respondent to the screening invitation or diagnosed before the screening invitation.

### The case–control study design

All areas where both screening and a cancer registry were active were included in the case–control study. The study base was the dynamic population of women aged 50–74 years resident in the selected areas between the year before the start of service screening and 2001 (i.e., all women who were resident in the areas for any period of time during the study period were eligible for the study).

Cases were defined as breast cancer deaths as reported in death certificates occurred not later than 31 December 2002 in the study base. To check for the information bias because of possible misclassification of cause of death, all deaths within breast cancer cases diagnosed in the study base were included in the study and a sensitivity analysis using deaths for whatever cause was performed.

Each case was matched to four controls using incidence–density sampling. The risk set for each case was defined as the women resident in the municipality in the subject's year of death. Controls were individually matched to the fatal breast cancer cases by area of residence and date of birth (±3 months). All controls had to be free of breast cancer up to the date of diagnosis of the matched case (the eligibility was assessed by linkage with the cancer registry).

Cases and controls were only enrolled if they were resident in their municipality from at least 2 years before the date of case diagnosis or from the screening programme start date.

### Definition of exposure

The service screening histories of cases and controls, including the date of their first invitation and the dates of all their screening tests following the invitation, if any, were extracted from the local, computerised, screening database. All and only mammography tests performed in service screening, that is, following an invitation to be screened, were included in the analysis, regardless of the actual test result.

The date of incidence reported in the cancer registry and the date of pseudodiagnosis for controls (i.e., the date of censoring the screening history for controls) were selected as the index dates for the classification of subjects as either invited or not-yet-invited and as either screened or unscreened.

Statistical modelling using microsimulation has been implemented by Connor *et al.* (2000) showing how estimates of mortality odds ratios are influenced by the choice of a time window in which controls are eligible to be screened. If a matched case is detected clinically, the date of pseudodiagnosis of the control should be defined as the date of diagnosis of the matched case (so excluding later screening test). Screen-detected breast cancer cases live without symptoms in a period corresponding to the diagnostic anticipation. Therefore detection at screening shortens a matched control's opportunity to be screened. On the basis of the results of microsimulation, Connor *et al* (2000) postulated that the definition of exposure to screening should include any screen up to the time the case would have been clinically diagnosed in the absence of screening. To compensate for the lead time owing to screen detection, the pseudodiagnosis date of the controls matched to each screen-detected case was postponed for 1 year by allowing the controls to be screened for a duration comparable with the preclinical detectable phase.

### Statistical analyses

We present firstly the analysis by allocation, that is, invited *vs* not-yet-invited women. In Italy, a large proportion of women waited for 2–4 years after the official programme start date before receiving their invitation to an appointment for the mammography test. This waiting time was an expected logistical and organisational consequence of enrolling the whole target population.

Our second analysis is the comparison of the mortality for breast cancer between screened and unscreened women (i.e., never-respondent plus not-yet-invited women). The inclusion of not-yet-invited women in the reference category with the never-respondent women would likely decrease the possible distortion of the comparison, but the combining of the two groups is not, *per se*, a guarantee of an unbiased estimate. In fact, respondent women might be different from never-respondent women because of the so-called ‘self-selection bias’: those screened have elected to be screened whereas those never screened (after the invitation) have refused the offer to be screened, thereby introducing a self-selection bias into the allocation of exposure (Cuzick *et al*, 1997).

Finally we compare screened with never-respondent women. The method proposed by Duffy *et al* (2002b), to correct for selection bias and thus obtain the effect of screening in potential attendees, was applied. The odds ratio corrected for selection bias was estimated with the following formula, where *p* is the attendance rate and *φ* is the crude uncorrected odds ratio and *Dr* is the mortality differential between never-respondent and not-yet-invited women: 
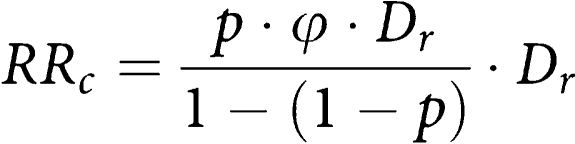
 To estimate the mortality differential *Dr*, the odds ratio of never-respondent to not-yet-invited women was calculated.

The odds ratios and 95% confidence intervals were calculated using conditional logistic regression retaining individual matching for birth cohort and area of residence. The statistics package used for the analyses was STATA 9.2.

## Results

A total of 2371 deaths within breast cancer cases diagnosed in the target population of women aged 50–74 years were included in the study. Four controls were matched to each case by date of birth and municipality of residence for a total of 9484 controls (1 : 4). A total of 1750 (73.8%) deaths were caused by breast cancer.

Patient characteristics and screening history by case–control status are presented in Table 1. The year of screening activation is different between programmes and it varies from the early 1990s in Tuscany and Piedmont to the late 1990s in Veneto. The average age at diagnosis/pseudodiagnosis was 62.3 years for cases and 62.2 years for controls. The 37.5 and 39.6% among cases and controls respectively had been invited to screening. Considering invited women only, 54.8 and 38.0% had never attended to screening among cases and controls respectively. Mean age at first screening was 59.2 for cases and 60.0 for controls.

Screen-detected cases accounted for 10.3% of breast cancer deaths, 6.6% were diagnosed in not screen-detected women with at least one screening test and 20.6% in never-respondent women. The remaining breast cancer deaths occurred among the not-yet-invited women. Among the case subjects who died for breast cancer, 173 (9.9%) were classified as early cancer at diagnosis (stage less than II) and 1301 (74.3%) were stage II+. The average age at death was 65.2 and average time from diagnosis to death was 2.9 years (range: <1–12 years), with mean times of 3.2 years for screen-detected breast cancer cases, 2.4 years for never-respondent women and 3.0 years for not-yet-invited women.

The logistic conditional estimate of the odds ratios for risk of breast cancer deaths comparing invited with not-yet-invited women was 0.75 (95% CI: 0.62–0.92), a 25% mortality reduction, and the odds ratio comparing screened with unscreened women was 0.50 (95% CI: 0.42–0.59) (Table 2). These odds ratios do not vary significantly by age group (homogeneity test: *P*=0.278 and *P*=0.196, respectively).

The odds ratios comparing screened with never-respondent women were performed including only cases and controls with at least one invitation to screening (*N* cases=657), to ensure that all subjects were comparable with respect to screening opportunity. The odds ratio of screened to never-respondent women was 0.46 (95% CI: 0.38–0.56), a 54% mortality reduction (Table 2). According to Duffy *et al* (2002b), the correction for selection bias brought to an estimate of the corrected odds ratio for screened to never-respondent women equal to 0.55 (95% CI: 0.36–0.85), a 45% mortality reduction, confirming the protective effect of screening for those women attending (Table 2). This corrected estimate has been obtained assuming 65% of attendance and estimating the mortality differential through the odds ratio of never-respondent to not-yet-invited women (1.11, 95% CI: 0.87–1.40).

We performed a sensitivity analysis including deaths within breast cancer cases for whatever cause. The probability of dying for all causes for invited *vs* not-yet-invited women was 0.83 (95% CI: 0.70–0.98) and for screened *vs* unscreened women was 0.63 (95% CI: 0.55–0.73).

## Discussion

Data from this study has been extracted from the Italian IMPACT database where all breast cancer cases have been classified by detection method and staged. The definition of the exposure to screening is based on computerised records of screening service. Breast cancer cases have been obtained from cancer registries (independent sources) and linked blind to the screening histories.

With a study size of 1750 cases and 7000 matched controls, the study has 90% power (*α*=0.05) to detect an 18% difference in mortality between invited and not-yet-invited women.

The results of this study show that service screening is associated with a 25% reduction in the probability of dying for breast cancer by allocation to screening invitation and with a 45% reduction when comparing screened with never-respondent women after correction for selection bias.

The analysis by allocation allows an estimation of how much the organised screening programme has added to the background use of mammography. It should be considered as equivalent to an intention-to-treat, non-randomised analysis (Selby, 1994). Invited women included never-respondent women. Only screened cases benefit from a screening programme, and therefore the estimate of the impact of service screening is conditioned by the rate of compliance to the invitation. According to this analysis, our study has shown a reduction of breast cancer mortality within the range expected on the basis of RCTs reported in the literature (IARC, 2002). The summary estimate of the breast cancer mortality reduction, after pooling the results from eight RCTs, is equal to 24% (31–17%) (Demissie *et al*, 1998).

The analyses by exposure to screening measures the benefit of screening among women who agree to be screened, and therefore the result may be affected by self-selection bias. We followed two different strategies to deal with this possible bias: the comparison of the mortality for breast cancer between screened and unscreened women, that is, never-respondent plus not-yet-invited, and the comparison of screened women with those never-respondent among the invited women corrected for self-selection (Duffy *et al*, 2002b). Both the estimates showed that service screening is effective in reducing breast cancer mortality by about 45–50% in women attending after invitation.

In other recent case–control screening studies, there has been a range of results from small (Elmore *et al*, 2005) to large benefit (Fielder *et al*, 2004; Allgood *et al*, 2008). Elmore's study assesses the efficacy of screening in a context where the screening is based on spontaneous access to mammographic facilities, and the subject's screening history has been extracted from medical records. The lack of a reduction in breast cancer mortality may be partly because of a differential misclassification of the status of screening exposure. In contrast, in the case–control studies where the aim is to evaluate service-screening programmes, only mammography tests performed following an invitation have been included in the analysis. The design of these studies is rather similar to ours; in UK the study by Allgood *et al* (2008) estimated a 65% reduction of breast cancer mortality in women attending screening, whereas the study by Fielder *et al* (2004) estimated a 38% reduction.

Screened and never-respondent women could have a different background access to mammography, as well as other differences related to the measure of the screening effect. To fully control this selection bias, we performed the analysis using the method proposed by Duffy *et al* (2002b). This method of correction for self-selection bias, which has been used in several case–control studies (Fielder *et al*, 2004; Allgood *et al*, 2008), makes the crucial assumption that the relative excess mortality for ‘non-compliers’ compared with a population not invited for screening is the same in the programme in question as in the RCTs (pooled estimate: 36%). The estimate of the relative mortality obtained from RCTs is too high for the Italian service-screening programme where the participation rate is lower and therefore the difference between compliers and non-compliers is not expected to be so large. From the information available in our dataset, we estimated a 11% relative excess mortality for never-respondent women and we used this internal estimate in the correction for self-selection bias.

Exposure is theoretically defined as screening (invitation or test) that takes place within that period before the time at which the case would have been diagnosed in the absence of screening. In empirical studies the time of diagnosis in the absence of screening for cancer is not observable and it is necessary, therefore, to use estimates of the average duration of the preclinical phase. We have assumed a 1-year time lag in exposure for controls matched to screen-detected cases. To evaluate the impact on the odds ratio of different time lags, we performed a sensitive analysis. The estimates of the odds ratios comparing invited with not-yet-invited women were 0.82 and 0.72 using time lags of 6 months and 1.5 years, respectively. The odds ratios comparing screened to unscreened women were 0.54–0.49, respectively.

We considered the possible information bias due to misclassification of cause of death (Gill and Horwitz, 1995). Extending the analysis to deaths for whatever cause, we obtained an estimate of 17% for the mortality reduction for invited women and 37% for screened women. A possible bias in the attribution of the cause of death cannot be excluded, but it seems implausible that it could explain all of the observed benefit.

We also considered the possibility that improvements in breast cancer treatment could have affected our estimate on the impact of screening, as far as the comparison of invited *vs* not-yet-invited is concerned. As a matter of fact the median year of diagnosis is 1 year more recent for invited with respect to not-yet-invited women. We do not think that important treatment improvements had occurred in so short a period. Furthermore, we previously showed in two of the areas participating in the IMPACT study that the improvement in survival rates were almost completely explained by stage distribution and not by increase of the survival rate by stage (Paci *et al*, 2005).

Few screen-detected breast cancer cases were dead at the end of follow-up (10.4% of the total deaths), and the majority of deaths were among the not-yet-invited and never-respondent women. This is the expected outcome if early diagnosis by mammographic screening is changing the probability of dying for breast cancer. Longer follow-up can confirm that service screening will continue to achieve a reduction of mortality for breast cancer.

In conclusion, the present study shows that the introduction of breast cancer screening programmes in Italy is associated with a reduction in breast cancer mortality, consistent with the results of randomised trials of mammographic screening and attributable to the additional impact of service screening over and above the background access to mammography.

## Figures and Tables

**Table 1:** Patient characteristics, screening history by case–control status

		**Cases**	**Controls**
Region, *N* (%)	Screening activation		
Emilia-Romagna	1995	784 (44.8)	3136 (44.8)
Piedmont	1992	418 (23.9)	1672 (23.9)
Tuscany	1990	454 (25.9)	1816 (25.9)
Umbria	1997	42 (2.4)	168 (2.4)
Veneto	1999	52 (3.0)	208 (3.0)
			
Mean (range) age at diagnosis or pseudodiagnosis		62.3 (50–74)	62.2 (49–75)
			
*Invitation status*, *N* (%)			
Not-yet-invited		1093 (62.5)	4228 (60.4)
Invited		657 (37.5)	2772 (39.6)
			
*Number of screening visits among invited, N* (%)			
0		360 (54.8)	1054 (38.0)
1		212 (32.3)	1123 (40.5)
2		52 (7.9)	397 (14.3)
3+		33 (5.0)	198 (7.1)
			
Mean (range) age at first screening		59.2 (50–71)	60.0 (49–71)
			
*Mode of detection, N* (%)			
Screen-detected		181 (10.3)	
Not screen-detected with at least 1 screening test		116 (6.6)	
Never respondent		360 (20.6)	
Not-yet-invited		1093 (62.5)	
			
*TNM stage, N* (%)			
Early (stage 0–I)		173 (9.9)	
Advanced (stage II+)		1301 (74.3)	
Unknown		276 (15.8)	
			
Mean (range) age at death		65.2 (50–85)	

**Table 2:** The odds ratios for risk of breast cancer death by screening history

	**No of cases/ controls**	**Odds ratio (95% CI)**
*Analysis by allocation*		
Not-yet-invited	1093/4228	1
Invited^a^	657/2772	0.75 (0.62–0.92)
		
*Analysis by screening status*		
Unscreened^b^	1453/5282	1
Screened	297/1718	0.50 (0.42–0.60)
		
*Analysis by screening status among invited women only*		
Never respondent	360/761	1
Screened	297/1307	0.46 (0.38–0.56)
Screened (self-selection corrected)		0.55 (0.36–0.85)

a Screened+never-respondent.

b Never-respondent+not-yet-invited.
