# Effectiveness of service screening: a case–control study to assess breast cancer mortality reduction

**DOI:** 10.1038/sj.bjc.6604779

**Published:** 2008-11-11

**Authors:** D Puliti, G Miccinesi, N Collina, V De Lisi, M Federico, S Ferretti, A C Finarelli, F Foca, L Mangone, C Naldoni, M Petrella, A Ponti, N Segnan, A Sigona, M Zarcone, M Zorzi, M Zappa, E Paci, the IMPACT Working Group

**Correction to:** *British Journal of Cancer* (2008) **99**, 423–427, doi:10.1038/sj.bjc.6604532

Owing to an error during typesetting, there was a mistake introduced in Duffy's formula in this paper when first published in *British Journal of Cancer* in July 2008.

The formula was contained within the ‘Materials and Methods’ section and should have appeared, thus:

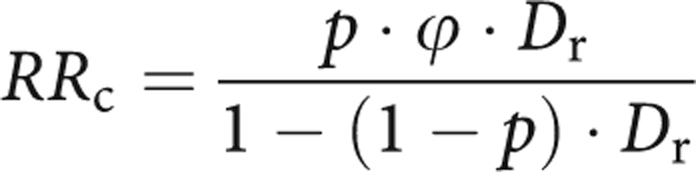


The publishers are happy to correct this mistake.

